# Book Review: “Blink: the power of thinking without thinking”

**DOI:** 10.3389/fpsyg.2015.01225

**Published:** 2015-08-19

**Authors:** Khushali Adhiya

**Affiliations:** Department of Psychology, Delhi Public SchoolAhmedabad, India

**Keywords:** power of thinking, thinking, thinking without thinking, judgment and decision making, thinking and reasoning

“It doesn't feel right!”

Ever caught yourself saying this? You probably then may have to lay your hands on understanding what drives that feeling inside you—a feeling that is completely contradictory to logic or decisions made on the basis of facts. BLINK gives you that understanding.

BLINK is written by psychotherapist Malcolm Gladwell, having four other popular contributions to his credit such as *The Tipping Point, Outliers*, etc. Gladwell's writing often centers around unforeseen implications of the social sciences' research. His books, articles, and speeches are received with great accolade and despite his critics having described him as “prone to oversimplification,” I found it quite to an advantage in his book BLINK. This book is considered one of his finest works by reviewers across the globe.

BLINK is a simple book, about how we actually think without thinking and the choices that follow such a thinking. The writer proves how the choices we make in an instant—in the blink of an eye- aren't that simple as they appear to be. So, this book reveals to you the mysteries of mind reading—an ability, which the reader realizes on completion of this book, lies within oneself.

The central focus of this book revolves around how we cognize the world around us, how we thin-slice (i.e., making a whole picture from just a gist), how rapid cognitions can be a lot better and productive than long but futile detailed analysis of situations, and thereby how these rapid cognitions sometimes lead us astray! The major contribution of this book comes around when Gladwell (2005) introduces us to our massive internal computer—the one that decides for us when we are unable to (consciously) and further on, how emotions play a huge, titanic role in our cognitions! Actually, that would be one of the most intriguing parts of this book.

BLINK is about human rapid cognition, a thinking that is a lot faster than we can realize it, and a thinking that operates quite mysteriously in comparison to our commonly used careful, planned, and thoughtful decision making. And that's the major hypothesis in this book. Along with this variable, through his extensive research and analysis, Gladwell (2005) introduces several other variables that are interdependent with rapid cognition—such as emotions, attitude, judgments, snap decisions, role of time, frugality of information, and role of past experiences in perception.

The author uses a very involving methodology to put forth his ideas; he utilizes a perfect mélange of questions (such as why do we fall for tall, dark men?), experiments very well researched and analyzed, and lots of case studies. This gigantic compilation, which seems exhaustive at one point, did after all do justice to the nature of the topic. I doubt there could have been a more involving way of putting across something that we can hardly decipher straightaway—the power of thinking without thinking!

For a sneak-peak, among the several answers Gladwell (2005) resolves through this book is one of the reasons why relationships fail, or rather what is it that indicates the downfall of a perfect-looking relationship. And as he unveils the shockers, you realize that so many times, we don't realize that we unknowingly give out non-verbal cues—and that is who we really are (not who we claim to be we are). This is where he presents to the reader the art of thin-slicing: making a judgment of the whole scenario from just a gist.

Thereon, Gladwell (2005) continues to provide innumerable such examples of thin-slicing in contexts such as speed-dating, tennis, gambling, military war games, malpractice suits, popular music, etc. The argument is how the unconscious thought overpowers a logically-thought decision and proves beneficial.

Gladwell (2005) explains that when we are thin-slicing or making our snap judgment, we've got a locked door i.e., our unconscious that makes those decisions for us, even before our conscious starts working on that information. Hence, the title “the power of thinking without thinking.” Our unconscious attitudes (and prejudices) play a deep role in our judgments and snap decisions.

It takes you in for a run when you read that snap decisions or split second decisions can take you astray. Gladwell (2005) deeply elaborates why and where can our mindreading abilities fail. He then spins your attention to the ability of problem solving—which involves a great deal of decision making. Some problem solving requires a flash of insight while some logic based-ones require explanations. However, trying to reflect or explain insightful problem solving can actually undermine the ability to insight!

Dijksterhuis (2004) in his recent research supported this possibility by reporting evidence that unconscious thinkers may make better decisions than conscious thinkers. On the same lines, in 2008 Gigerenzer discussed evidence of people often using reasoning “shortcuts” in their decision-making (Gigerenzer, 2008). He called them “heuristics” and spoke of intuition. [However, (Gladwell, 2005) did never intend to call his analysis an “intuition.” He refers to unconscious thought as the first 2 s—a thinking that moves a little faster and little more mysteriously]. On a third paradigm, Payne et al. (2008) argued that understanding the choice environment and interactions ought to be considered while considering how to approach complex choice problems.

Recently, these research points were criticized as possibly overrating the contribution of strictly unconscious processes to behavioral control (Newell and Shanks, 2014). While Gigerenzer and Dijksterhuis evince reasoning to be either conscious or unconscious, Gladwell (2005) demonstrates them as a process wherein the unconscious thought dominates for the first 2 s.

Gladwell (2005) substantiates that “truly successful thinking relies on a balance between deliberate and instinctive thinking,” which fits with the recent current conclusions of Nordgen et al. (2011).

BLINK is highly suitable for readers who crave to understand the complexities of human mind and decisions. Patience is a necessity and passion a prerequisite to absorb the hidden truths about your own mind while you read BLINK!

## Conflict of interest statement

The author declares that the research was conducted in the absence of any commercial or financial relationships that could be construed as a potential conflict of interest.
