# Corrigendum: Book Review: “Blink: the power of thinking without thinking”

**DOI:** 10.3389/fpsyg.2017.01334

**Published:** 2017-09-14

**Authors:** Khushali Adhiya

**Affiliations:** Department of Psychology, Delhi Public School Ahmedabad, India

**Keywords:** power of thinking, thinking, thinking without thinking, judgment and decision making, thinking and reasoning

The sentence

BLINK is written by a psychotherapist Malcolm Gladwell, having four other popular contributions to his credit such as *The Tipping Point, Outliers*, etc.

should be

BLINK is written by a journalist Malcolm Gladwell, having four other popular contributions to his credit such as *The Tipping Point, Outliers*, etc.

The authors apologize for this error and state that this does not change the scientific conclusions of the article in any way.

## Conflict of interest statement

The author declares that the research was conducted in the absence of any commercial or financial relationships that could be construed as a potential conflict of interest.

